# Peripheral blood toll-like receptor 4 correlates with rapid virological response to pegylated-interferon and ribavirin therapy in hepatitis C genotype 1 patients

**DOI:** 10.1186/s12876-016-0492-6

**Published:** 2016-07-25

**Authors:** Chuan-Mo Lee, Tsung-Hui Hu, Sheng-Nan Lu, Jing-Houng Wang, Chao-Hung Hung, Chien-Hung Chen, Yi-Hao Yen

**Affiliations:** 1 Division of Hepatogastroenterology, Department of Internal Medicine, Kaohsiung Chang Gung Memorial Hospital, 123 Ta Pei Road, Niao Sung Dist. 833, Kaohsiung City, Taiwan; 2 School of Medicine, College of Medicine, Chang Gung University, Taoyuan, Taiwan

**Keywords:** Hepatitis C virus, Toll-like receptor, Rapid virological response

## Abstract

**Background:**

Toll-like receptors (TLRs) are effectors of the innate immune system that are able to recognize hepatitis C virus (HCV) and give rise to an immune response. Failure of interferon (IFN)-α-based treatment is related to host immunity. Therefore, we sought to study the clinical importance of TLRs in HCV genotype 1 patients who received pegylated IFN (PEG-IFN) plus ribavirin (RBV) therapy.

**Methods:**

We enrolled 79 treatment-naïve patients with HCV genotype 1. Patients completed a 24- to 48-week course of response-guided therapy. Peripheral blood monocyte (PBMC) expression of mRNA for TLRs 2, 3, 4, 7, and 9 was quantified by real-time PCR before therapy. TLR mRNA expression is shown as a log ratio relative to GAPDH mRNA (log 2 ^-(∆Ct)^).

**Results:**

Forty-five patients (57.0 %) showed a rapid virological response (RVR). Univariate analysis revealed that TLR 2, 3, 4, 7, and 9 were significantly lower in the RVR group than in the non-RVR group (*P* = 0.001, 0.014, < 0.001, 0.008, and 0.001, respectively). Multivariate analysis revealed that TLR 4 < −2 log (OR: 7.17, 95 % CI: 1.70–30.34, *P* = 0.007) was an independent predictor for RVR. In addition, levels of TLR 2, 3, 4, 7, and 9 were positively correlated with HCV viral load (*P* = 0.009, 0.013, < 0.001, 0.007, and 0.001, respectively).

**Conclusions:**

A low level of TLR 4 mRNA in PMBCs was correlated with RVR, which indicates that TLR4 may play a critical role in HCV recognition and activation of innate immunity. TLR expression levels were correlated with HCV viral load, indicating that TLR activation upon exposure to HCV may subsequently limit HCV replication.

**Electronic supplementary material:**

The online version of this article (doi:10.1186/s12876-016-0492-6) contains supplementary material, which is available to authorized users.

## Background

Hepatitis C virus (HCV) is a blood-borne, hepatotrophic virus that establishes a chronic HCV infection in up to 85 % of cases [1]. With an estimated 2 % of the global population infected with HCV [2], which carries the potential for chronic infection leading to cirrhosis, end-stage liver diseases, and hepatocellular carcinoma (HCC), it poses a considerable health risk [3–6].

Non-alcoholic fatty liver disease (NAFLD) is now increasingly being recognized as a cause of end-stage liver disease and is associated with increased rates of HCC, liver transplantation, and death [7–9]. Current population based prevalence of NAFLD is approximately 70 % in people with type 2 diabetes mellitus (DM) [10]. Further, a recent study showed that liver transplant recipients with non-alcoholic steatohepatitis (NASH) have a higher risk of de novo post-transplant DM. This suggests the presence of an underlying metabolic disorder beyond fatty liver that may be causative for both NASH and type 2 diabetes [11].

Infection with HCV genotype 1, the prevalent genotype in Taiwan, Japan, and Southern and Eastern Europe [12–16], is predictive of a poor response to interferon (IFN)-based therapy. The sustained virological response (SVR) is 50–80 % following combination therapy with pegylated interferon (peg-IFN) plus ribavirin (RBV) for 48 weeks [17–20].

The European Association for the Study of the Liver (EASL) and the American Association for the Study of Liver Diseases (AASLD) guidelines [21, 22] both suggest direct antiviral agents (DAAs) as the first line of therapy for patients infected with genotype 1. However, the SVR rate can reach 76 % after 48 weeks of peg-IFN plus RBV therapy in Taiwanese patients with genotype 1 [20], and peg-IFN plus RBV therapy remains the standard of care in Taiwan. Therefore, it is crucial to determine the mechanism of treatment failure for IFN-based therapy in Taiwan.

Host immunity is an important factor that is related to the failure of IFN-α-based treatment. The innate immune system is particularly relevant in viral infections [23]. Toll-like receptors (TLRs), as effectors of the innate immune system, are activated immediately upon exposure to infectious agents and may subsequently limit replication of infectious agents [24].

TLRs belong to a family of cell receptors that are present on mammalian cells, and that are able to recognize several pathogen-associated molecular patterns (PAMPs) present on microbes [25]. Various viral components (RNA, viral proteins, and intact virions) can be recognized as PAMPs by the immune system; recognition can give rise to an immune response [25], including up-regulation of IFN-α production [23]. This may induce enhanced expression of IFN-α-inducible genes, most of which perform important antiviral and immune regulatory functions. In vitro studies have indicated that, of the 11 human TLRs identified so far, TLR 2, 3, 4, 7, and 9 recognize specific HCV viral components as PAMP ligands [26–33].

Whether TLR expression is associated with a virological response in patients infected with HCV genotype 1 remains unclear. In this study, we investigated whether TLR expression on peripheral blood monocytes (PBMCs) is associated with virological responses to peg-IFN plus RBV therapy in patients infected with HCV genotype 1.

## Methods

### Patients

We enrolled 79 treatment-naïve patients with HCV genotype 1, who then completed a 24- to 48-week course of response-guided therapy (RGT) with peg-IFN plus RBV. The RGT received was simply part of standard care in Taiwan and was funded by the Bureau of National Health Insurance, Department of Health, Taiwan. RGT was performed as follows: 24-week therapy for patients with rapid virological response (RVR) (defined as undetectable HCV RNA at week 4); 48-week therapy for patients with early virological response (EVR) (defined as detectable HCV RNA at week 4 and a > 2-log decrease in HCV RNA at week 12); therapy was stopped at week 12 for patients with a null response (defined as a > 2-log decrease in HCV RNA at week 12). PegIFN α-2b (Peg-Intron, Schering-Plough Corporation, Kenilworth, NJ) at 1.5 μg/kg or PegIFN α-2a (Pegacys, Roche, Basel, Switzerland) at 180 μg was given subcutaneously once weekly. RBV was given at 1000 mg/day for patients with a body weight of ≤ 75 kg and 1200 mg/day for those > 75 kg. Forty five patients achieved RVR, 34 patients did not. The characteristics of non-RVR group patients were as follow: there were 19 male and 15 female, the median age was 53.4 years, the median body mass index (BMI) was 26.4 Kg/m^2^, the median Alanine Aminotransferase (ALT) was 84.5 IU/L, 28 patients had high viral load (≥ 4 × 10^5^ IU/ml), 6 patients had liver crrhosis, 24 patients had rs12979860 CC genotype, and 24 patients with good drug adherence.

Patients who were positive for anti-HCV antibody, assessed by third-generation enzyme-linked immunosorbent assay, were diagnosed with chronic hepatitis C; the diagnosis was confirmed by detection of serum HCV-RNA. Patients were excluded if they tested positive for serum hepatitis B surface antigens, anti-HIV antibodies, or exhibited other causes of hepatocellular injury (e.g., any history of alcoholism, autoimmune hepatitis, primary biliary cirrhosis, or treatment with hepatotoxic drugs).

HCV RNA was qualitatively detected using a PCR-based assay (Cobas Amplicor Hepatitis C Virus Test, version 2.0, Roche Molecular Systems, Branchburg, NJ, USA), which has a lower limit of detection of approximately 50 IU/mL. HCV RNA was quantified by real-time PCR-based assay (COBAS AmpliPrep/COBAS TaqMan HCV Test, Roche), with a dynamic range of 43 to 69,000,000 IU/ml. Genotyping of HCV was performed using a Siemens Diagnostics Versant HCV Genotype Assay. SVR was defined as serum HCV RNA undetectable at week 24 after treatment.

### PBMC preparation and RNA extraction

PBMCs were isolated by Ficoll-Hypaque density gradient centrifugation from the peripheral venous blood of the subjects before antiviral therapy. Total cellular RNA was extracted using the TRizol Reagent (Invitrogen, Carlsbad, CA) in accordance with the manufacturer’s instructions. RNA yield and purity were determined by measuring the absorbance at 260/ 280 nm on a NanoDrop spectrophotometer (Thermo Fisher Scientific, Waltham, Massachusetts, USA).

### mRNA expression of TLR genes in PBMCs

To quantify mRNA expression of TLR genes (including TLR 2, 3, 4, 7, and 9) in PBMCs, we performed quantitative reverse transcription polymerase chain reactions (QRT-PCR) with a LightCycler 480 system (Roche Diagnostics GmbH, Mannheim, Germany). The reagent mixture was prepared according to the protocol provided by the manufacturer (Roche Diagnostics GmbH, Mannheim, Germany). Total RNA (2 μg) was extracted from PMBCs and cDNA was generated using an oligo(dT)_18_ primer (GeneMark Taiwan). PCR was performed in a 10-μl reaction mixture containing 2× LightCycler 480 SYBR Green I Master mix (5 μl) (Roche Diagnostics GmbH, Mannheim, Germany), forward and reverse primers (0.5 μM each), and cDNA (1 μl). The conditions for amplification were as follows: one cycle of 95 °C for 10 min and 40 cycles of 95 °C for 10 s, 60 °C for 12 s, and 72 °C for 20 s. After amplification was completed, a final melting-curve analysis was performed by denaturation at 95 °C, re-annealing at 65 °C for 1 min, and then slow heating (0.1 °C/s) to 95 °C to determine the product-specific melting temperature. The reaction chamber was then cooled to 40 °C for 30 s prior to opening the chamber to remove the plate, as recommended by the manufacturer. Data were analyzed using the LightCycler Software 1.5 (Roche Diagnostics GmbH, Mannheim, Germany). The threshold cycle value (Ct) was determined using the following equation: ΔCt = Ct (target gene) - Ct (GAPDH). The expression of target genes in each sample was tested in triplicate. mRNA expression of target genes is shown as a log ratio relative to GAPDH mRNA (log 2 ^-(∆Ct)^).

### Genetic variation in interleukin (IL)28B

Patient IL28B genotypes ere determined with ABI TaqMan SNP genotyping assays (Applied Biosystems, Foster City, CA) and with predesigned commercial genotyping assays (ABI assay ID: C__11710096_10). Briefly, PCR primers and two allele-specific probes were designed to detect a specific Single-nucleotide polymorphism (SNP) target. PCR reactions were performed in 96-well microplates with a StepOnePlus™ Real-Time PCR System (Applied Biosystems). Allele discrimination was achieved by fluorescence detection using the StepOne™ Software v2.1.

### Statistical analysis

We analyzed differences between virological responders and non-responders using the chi-square test and Student’s *t*-test. Independent factors that may have influenced the response to combination therapy were identified using stepwise multiple logistic regression analysis. We used Pearson’s correlation coefficient analysis to evaluate the correlation between mRNA expression of TLRs and baseline clinical variables. A *P*-value of <0.05 was considered statistically significant.

## Results

### Comparison of baseline features in patients with RVR and non-RVR

Forty-five patients (57 %) achieved RVR. The proportion of patients with a low viral load (< 4 × 10^5^ IU/ml), rs12979860 CC genotype, and rs8099917 TT genotype was higher in the RVR group than in the non-RVR group. In addition, the median body mass index (BMI) was lower and mRNA expression of TLR 2, 3, 4, 7, and 9 was lower in the RVR group, compared with the non-RVR group.

There were no significant differences between the RVR and non-RVR patient groups in terms of gender, ALT levels, rates of adherence to treatment, or the proportion of patients with liver cirrhosis (Table 1).

**Table 1:** Comparisons of baseline features of patients with rapid virological response (RVR) and those with non-RVR

Variables	RVR (*N* = 45)	Non-RVR (*N* = 34)	*P* value
Sex (M:F)	23:22	19:15	0.674
Age (median,IQR)	60.5 (52.9 ~ 67.8)	53.4 (43.9 ~ 63.0)	0.003
BMI, Kg/m^2^ (median,IQR)	23.5 (21.8 ~ 24.7)	26.4 (23.0 ~ 29.4)	0.001
ALT, IU/L(median,IQR)	71.0 (48.0 ~ 138.5)	84.5 (56.3 ~ 115.5)	0.801
HCV viral load (≥ vs < 4x10^5^ IU/ml)	17:28	28:6	< 0.001
Liver cirrhosis (yes vs. no)	4:41	6:28	0.313
> 80 % adherence (yes vs. no)	32:13	24:10	0.960
rs12979860 (CC vs. CT + TT)	43:2	24:10	0.002
rs 8099917 (TT vs. GG + GT)	44:1	25:9	0.002
TLR2 mRNA (median,IQR)	−1.36 (−2.52 ~ 0.76)	0.82 (0.55 ~ 0.96)	0.001
TLR3 mRNA (median,IQR)	−2.66 (−3.13 ~ −1.28)	−1.47 (−1.90 ~ −1.27)	0.014
TLR4 mRNA (median,IQR)	−1.85 (−2.53 ~ 0.57)	0.66 (0.31 ~ 0.86)	< 0.001
TLR7 mRNA (median,IQR)	−2.41 (−3.19 ~ −0.07)	−0.77 (−1.18 ~ −0.57)	0.008
TLR9 mRNA (median,IQR)	−2.44 (−3.17 ~ −1.01)	−0.98 (−1.26 ~ −0.84)	0.001

*BMI* body mass index, *ALT* alanine aminotransferase, *TLR* toll like receptor, *TLR* mRNA expressions were shown as log ratios relative to GAPDH mRNA (log 2 ^-(∆Ct)^). > 80 % adherence: patients who received more than 80 % of standard dose and duration

### Multivariate analysis of pretreatment factors associated with RVR

Stepwise multiple logistic regression analysis revealed that mRNA expression of TLR 4 < −2 log (odds ratio (OR), 7.17; 95 % confidence interval (CI), 1.70–30.34; *P* = 0.007), a viral load <4 × 10^5^ IU/ml (OR, 7.08; 95 % CI, 1.17–30.09; *P* = 0.008), and rs 8099917 TT genotype (OR, 38.8; 95 % CI, 2.65–568.63; *P* = 0.008) were independent predictors for RVR.

### Comparison of baseline features in patients with SVR and non-SVR

Four patients who completed the treatment but not the follow-up were excluded. The remaining 75 patients were analyzed. Of these 75 patients, 45 (60 %) achieved SVR. The proportion of males and the rate of liver cirrhosis were both lower in the SVR group than in the non-SVR group. The proportion of patients with RVR, rs 12979860 CC genotype, and rs 8099917 TT genotype were higher in the SVR group than in the non-SVR group. There were no significant differences between the SVR and non-SVR patient groups in terms of TLR expression (Table 2).

**Table 2:** Comparisons of baseline features of patients with sustained virological response (SVR) and those with non-SVR

Variables	SVR (*N* = 45)	Non-SVR (*N* = 30)	*P* value
Sex (M:F)	19:26	20:10	0.038
Age (median,IQR)	59.7 (51.1 ~ 67.4)	56.6 (45.9 ~ 64.7)	0.176
BMI, Kg/m^2^ (median,IQR)	23.9 (22.4 ~ 27.1)	23.7 (21.4 ~ 27.8)	0.787
ALT, IU/L(median,IQR)	84.0 (52.0 ~ 134.5)	77.0 (44.8 ~ 14.8)	0.423
Viral load (≥ vs < 4 × 10^5^ IU/ml)	25:20	19:11	0.503
RVR (yes vs. no)	31:14	10:20	0.002
Liver cirrhosis (yes vs. no)	2:43	8:22	0.012
> 80 % adherence (yes vs. no)	32:13	21:9	0.918
rs12979860 (CC vs CT + TT)	43:2	20:10	0.002
rs 8099917 (TT vs GG + GT)	45:0	20:10	< 0.001
TLR2 mRNA (median,IQR)	−1.07 (−4.05 ~ −0.74)	−0.96 (−3.39 ~ −0.67)	0.304
TLR3 mRNA (median,IQR)	−3.39 (−4.53 ~ −2.86)	−3.28 (−4.10 ~ −2.90)	0.607
TLR4 mRNA (median,IQR)	−1.24 (−4.05 ~ −0.96)	−1.02 (−3.54 ~ −0.78)	0.067
TLR7 mRNA (median,IQR)	−2.76 (−4.53 ~ −2.19)	−2.42 (−4.18 ~ −2.20)	0.545
TLR9 mRNA (median,IQR)	−2.85 (−4.57 ~ −2.59)	−2.62 (−4.12 ~ −2.47)	0.124

*RVR* rapid virological response, *BMI* body mass index, *ALT* alanine aminotransferase, *TLR* toll like receptor. TLR mRNA expression levels were shown as log ratios relative to GAPDH mRNA (log 2 ^-(∆Ct)^)

### Multivariate analysis of pretreatment factors associated with SVR

Stepwise multiple logistic regression analysis revealed that non-liver cirrhosis (OR, 6.94; 95 % CI, 1.22–40.0; *P* = 0.029), rs 12979860 CC genotype (OR, 6.53; 95 % CI, 1.18–36.01; *P* = 0.031), and RVR (OR, 3.06; 95 % CI, 1.01–9.28; *P* = 0.048) were independent predictors for SVR.

### Correlation between mRNA expression of TLRs and baseline clinical features

mRNA expression of TLR 2, 3, 4, 7, and 9 was correlated with HCV viral load, but was not correlated with ALT levels or liver cirrhosis (Table 3).

**Table 3:** Correlation between TLR mRNA expression levels and baseline clinical factors (*N* = 79)

	TLR2		TLR3		TLR4		TLR7		TLR9	
	*R* value	*P* value	*R* value	*P* value	*R* value	*P* value	*R* value	*P* value	*R* value	*P* value
HCV RNA	0.292	0.009	0.277	0.013	0.386	< 0.0001	0.301	0.007	0.354	0.001
ALT	−0.126	0.269	0.046	0.688	−0.009	0.934	0.097	0.393	−0.05	0.663
Cirrhosis	−0.124	0.278	0.087	0.447	−0.024	0.832	0.014	0.901	−0.901	0.425

Spearman correlation. HCV RNA (log IU), *ALT* alanine aminotransferase (U/dl). Cirrhosis:yes vs. no. TLR mRNA expression levels were shown as log ratios relative to GAPDH mRNA (log 2 ^-(∆Ct)^)

## Discussion

In this study, we found that low TLR4 mRNA expression in PBMCs was associated with RVR in patients with HCV genotype 1 who received peg-IFN and RBV therapy. Univariate analysis also revealed that TLR 4 expression is lower in the SVR group than in the non-SVR group (*P* = 0.067), although it is not statistically significant. The case number in our study is limited, may be more case number is needed to reach statistically significant.

TLR4, a lipopolysaccharide-receptor, plays a critical role in pathogen recognition and activation of innate and adaptive immunity. Ten et al. studied the release of soluble TLR2 and TLR4 in plasma of 394 patients with infections (infectious mononucleosis, measles, respiratory tract infections, bacterial sepsis and candidemia) or non-infectious inflammation (Crohn’s disease, gout, rheumatoid arthritis, autoinflammatory syndromes and pancreatitis). They found that soluble TLR4 had a similar capacity for differentiating infectious and non-infectious inflammation compared to C reactive protein (CRP), and suggest the possibility to use soluble TLRs as diagnostic tool in inflammatory conditions [34].

Machida et al. reported that HCV non-structural 5A (NS5A) protein, which plays a potential role in resistance to IFN-α treatment, transactivates the TLR4 promoter in vitro, resulting in increased transcription of TLR4 [29].

He et al. enrolled 15 HCV infected patients who had received a 48-week treatment with peg-IFN and RBV. They found that baseline PBMC TLR 2 and 3 mRNA levels were significantly higher in patients with SVR than in non-responders. They suggested that TLR 2/3, which was up-regulated in PBMCs, might act as an adjuvant receptor, increasing not only the sensitivity to HCV PAMPs but also the sensitivity to INF-α treatment, and subsequently increased effective antiviral responses [35]. However, the number of cases examined was small.

Yuki et al. reported that low hepatic TLR3 expression was a predictor of SVR to peg-IFN plus RBV in patients with HCV genotype 1 [36]. TLRs are known to be intrahepatic interferon stimulated genes (ISGs) and these results were consistent with previous studies which found that pre-activation of intra-hepatic ISGs was associated with non-response in HCV-infected patients [37–40]. Activation of the endogenous IFN system in HCV-infected patients was ineffective in clearing the infection and even impeded the response to therapy, most likely by inducing a refractory state in the IFN signaling pathway [37–40]. However, analyzing gene expression in liver biopsy specimens requires an invasive procedure.

In this study, we found that mRNA expression of TLR 2, 3, 4, 7, and 9 was correlated with HCV viral load. TLR activation upon exposure to HCV may subsequently limit HCV replication [25, 26, 28, 31, 32]. However, HCV can escape host immunity via several methods and sustain a chronic infection [30, 41, 42]. These interactions between HCV and TLRs may be one reason for the correlation between TLR expression and HCV viral load observed in our study.

## Conclusion

Low PMBC TLR 4 levels were correlated with RVR, which indicates that TLR4 may play a critical role in HCV recognition and activation of innate and adaptive immunity. mRNA expression of TLRs was correlated with HCV viral load, which indicates that TLR activation upon exposure to HCV may subsequently limit HCV replication.

## Abbreviations

AASLD, American Association for the Study of Liver Diseases; ALT, Alanine Aminotransferase; BMI, body mass index; CRP, C reactive protein; Ct, threshold cycle value; DAAs, direct antiviral agents; DM, diabetes mellitus; EASL, European Association for the Study of the Liver; EVR, early virological response; HCC, hepatocellular carcinoma; HCV, hepatitis C virus; IFN, interferon; ISGs, interferon stimulated genes; NAFLD, Non-alcoholic fatty liver disease; NASH, non-alcoholic steatohepatitis; NS5A, non-structural 5A; PAMPs, pathogen-associated molecular patterns; PBMCs, peripheral blood monocytes; PEG-IFN, pegylated interferon; QRT-PCR, quantitative reverse transcription polymerase chain reactions; RBV, ribavirin; RGT, response-guided therapy; RVR, rapid virological response; SVR, sustained virological response; TLRs, Toll-like receptors

## Additional file

Additional file 1: Raw data of these population. (XLS 79 kb)
